# Supplementary material for: Robust performance of culture, real-time PCR, and genomic approaches for shigella serotyping in a pediatric surveillance cohort
Source: PLoS Negl Trop Dis. 2026 Aug 26;20(8):e0014668. doi: 10.1371/journal.pntd.0014668 (PMC13614632; doi:10.1371/journal.pntd.0014668)
Supplement: Supplementary file 1 [file pntd.0014668.s001.docx]

**S1 Table.** Primers and probes used for Shigella serotyping, including gene targets and sequences.

| **Gene target** | **Forward primer** | **Reverse primer** | **Probe** |
| --- | --- | --- | --- |
| ***Shigella flexneri*** | | | |
| *gtrI* | AAATATGCCTCCATACAATTG | AGCATATGTATTAAACAATCAGCA | GCTGTTAGCAACATCCGGTTCAAC |
| *gtrIc* | ACCTTAGGTTCAAATGGGTTAC | GAAATAGCCGTCTCTCGAATA | TGTTTTCACATTTAGTATTCCAAC |
| *gtrII* | CAAACGACTCAGGAAATATGC | AATTCATAAATGCAACCATCCT | CTCCATGAGCGCAGACACTTTTG |
| *gtrIV* | TCTCACATGATGGCACATTA | CCTAAGATCAAATGTGTGTGTGA | TTTATACCCTGAAGGAAAATTTCAG |
| *gtrV* | TTATTAATGTCATCGTCCATCC | CCACTCCCAGATTACGG | GCAGGGTTGAACTTGAAAGAATACGAT |
| *gtrX* | AGCACCACATCAAAAATCTTC | CATACAATGATAAATACCAGTGAGCATT | TATATTTAATTTGCATGCCCGGGC |
| *oac* | GCATAAGAGCAACTGCTTTG | CGCGTAGTGGTGACTG | ACGGCAAGGCTTGTGGCA |
| *wzx6* | GAGCGATCATTTCAACTTCA | TACAACATGATTCGCGTTAATGT | CGGTAATTCTAACTATATTGGGCTTG |
| ***Shigella sonnei*** | | | |
| *Rhs* | CAGTGGCTGGCATATTTCGA | TGCTATACTCCAATCCTGTCTGTTTT | CTGACGCCGGAGTAGAT |
| *pm* | TGCCGCTAAAATCCTTCTGT | GCGTACGACGAAAGGAAAAA | AAGTTATTGATTCCGCCC |
| ***Shigella* spp.** | | | |
| *ipaH* | CCTTTTCCGCGTTCCTTGA | CGGAATCCGGAGGTATTGC | CGCCTTTCCGATACCGTCTCTGCA |
| **Internal control** | | | |
| PhHV *gb* | GGGCGAATCACAGATTGAATC | GCGGTTCCAAACGTACCAA | TATGTGTCCGCCACCATCT |
