# Supplementary material for: Robust performance of culture, real-time PCR, and genomic approaches for shigella serotyping in a pediatric surveillance cohort
Source: PLoS Negl Trop Dis. 2026 Aug 26;20(8):e0014668. doi: 10.1371/journal.pntd.0014668 (PMC13614632; doi:10.1371/journal.pntd.0014668)
Supplement: Supplementary file 3 [file pntd.0014668.s003.docx]

**S3 Table .** Stool based real-time PCR serotype assignment.

| **PID** | **Serotype** |
| --- | --- |
| 6100216 | S.sonnei |
| 6100225 | S.sonnei |
| 6100305 | S.flexneri 2a |
| 6100331 | Untyped |
| 6100393 | S.flexneri 2a |
| 6100474 | Untyped |
| 6100495 | S.sonnei |
| 6100572 | S.flexneri 1b |
| 6100585 | S.flexneri 2a |
| 6100609 | S.sonnei |
| 6100611 | S.sonnei |
| 6100653 | S.flexneri 2a |
| 6100712 | S.sonnei |
| 6100773 | S.flexneri 2a |
| 6100845 | S.sonnei |
| 6100850 | Untyped |
| 6100861 | S.sonnei |
| 6100971 | S.flexneri 2a |
| 6101001 | S.flexneri 1b |
| 6101064 | S.sonnei |
| 6200092 | S.flexneri 2a |
| 6200136 | S.flexneri 4b |
| 6200160 | S.flexneri 4b |
| 6200212 | S.sonnei |
| 6200235 | Untyped |
| 6200321 | S.flexneri 2a |
| 6200363 | Untyped |
| 6200434 | S.sonnei |
| 6200465 | Untyped |
| 6200490 | S.flexneri 2b |
| 6200532 | S.flexneri 2a |
| 6200711 | S.flexneri 2a |
| 6200905 | S.flexneri 3a |
| 6200910 | S.sonnei |
| 6200968 | S.flexneri 1b |
| 6201011 | S.sonnei |
| 6201027 | S.flexneri 2b |
| 6201075 | S.sonnei |
| 6201121 | S.flexneri 1a |
| 6201139 | S.flexneri 1a |
| 6201142 | S.sonnei |
| 6201188 | S.sonnei |
| 6201195 | Untyped |
| 6201214 | Untyped |
| 6201238 | Untyped |
| 6201277 | S.sonnei |
| 6201371 | S.flexneri 1b |
| 6201405 | S.flexneri 1b |
| 6201422 | S.sonnei |
| 6201446 | S.sonnei |
| 6201468 | S.flexneri 2a |
| 6201479 | S.flexneri 2a |
| 6201493 | S.flexneri 1a |
| 6201529 | S.flexneri 2a |
| 6201565 | Untyped |
| 6201635 | S.flexneri 2a |
| 6201699 | Untyped |
| 6201702 | Untyped |
| 6201725 | Untyped |
| 6201778 | Untyped |
| 6201949 | Untyped |
| 6201960 | S.sonnei |
| 6201972 | S.sonnei |
| 6201985 | S.sonnei |
| 6201997 | S.sonnei |
| 6202074 | S.sonnei |
| 6202095 | S.flexneri 2a |
| 6202182 | Untyped |
| 6202267 | Untyped |
| 6202564 | S.sonnei |
| 6202607 | S.sonnei |
| 6300154 | S.flexneri 4b |
| 6300219 | S.flexneri 4b |
| 6300237 | S.flexneri 2a |
| 6300255 | S.sonnei |
| 6300262 | S.sonnei |
| 6300336 | S.sonnei |
| 6300397 | S.flexneri 2a |
| 6300459 | Untyped |
| 6300471 | S.sonnei |
| 6300521 | Untyped |
| 6300633 | Untyped |
| 6300657 | Untyped |
| 6300738 | Untyped |
| 6300882 | Untyped |
| 6300894 | S.sonnei |
| 6300918 | S.flexneri 2b |
| 6301014 | S.flexneri 2a |
| 6301023 | S.flexneri 2b |
| 6301092 | S.flexneri 2a |
| 6301185 | S.flexneri 2b |
| 6301220 | S.flexneri 2b |
| 6301356 | S.flexneri 4a |
| 6301465 | S.flexneri 2a |
| 6301476 | Untyped |
| 6301567 | S.sonnei |
| 6301580 | S.sonnei |
| 6301766 | S.sonnei |
| 6301775 | S.sonnei |
| 6301782 | S.sonnei |
| 6301794 | S.sonnei |
| 6301802 | S.sonnei |
| 6301816 | S.sonnei |
| 6301840 | Untyped |
| 6301869 | S.flexneri 1b |
| 6301878 | Untyped |
| 6301884 | S.flexneri 2a |
| 6301922 | S.flexneri 2a |
| 6301987 | S.sonnei |
| 6302051 | S.flexneri 4a |
| 6302085 | S.flexneri 1b |
| 6302161 | S.sonnei |
| 6302177 | S.flexneri 2a |
| 6302189 | S.flexneri 2a |
| 6302192 | S.flexneri 2a |
| 6302200 | S.flexneri 2a |
| 6302217 | S.flexneri 2a |
| 6302221 | Untyped |
| 6302301 | S.flexneri 2a |
| 6302364 | Untyped |
| 6302402 | Untyped |
| 6302528 | S.sonnei |
| 6302543 | S.sonnei |
| 6302581 | S.sonnei |
| 6302698 | S.flexneri 3a |
| 6302710 | S.flexneri 2a |
| 6302722 | S.sonnei |
| 6302746 | Untyped |
| 6302852 | S.flexneri 1b |
| 6302890 | S.flexneri 1a |
| 6302909 | S.flexneri 2a |
| 6302953 | S.flexneri 3a |
| 6302966 | S.flexneri 3a |
| 6303025 | Untyped |
| 6303069 | S.flexneri 2a |
| 6303084 | S.flexneri 2a |
| 6303198 | Untyped |
| 6303318 | S.sonnei |
| 6303352 | S.flexneri 2a |
| 6303408 | S.flexneri 2a |
| 6303692 | S.sonnei |
| 6303728 | S.sonnei |
| 6400133 | S.sonnei |
| 6400169 | S.sonnei |
| 6400229 | Untyped |
| 6400330 | S.flexneri 1b |
| 6400366 | S.flexneri 1b |
| 6400401 | S.flexneri 2a |
| 6400538 | S.sonnei |
| 6400545 | S.sonnei |
| 6400646 | S.flexneri 2a |
| 6400654 | S.flexneri 1b |
| 6400668 | Untyped |
| 6400687 | S.flexneri 2a |
| 6400721 | S.sonnei |
| 6400742 | S.flexneri 2a |
| 6400808 | Untyped |
| 6400919 | S.flexneri 3a |
| 6401088 | S.sonnei |
| 6401194 | S.flexneri 2a |
| 6500027 | S.flexneri 2a |
| 6500142 | S.flexneri 4a |
| 6500174 | Untyped |
| 6500292 | S.flexneri 2a |
| 6500332 | S.sonnei |
| 6500380 | Untyped |
| 6500405 | S.sonnei |
| 6500410 | S.sonnei |
| 6500446 | Untyped |
| 6500493 | S.flexneri 2a |
| 6500583 | S.flexneri 2b |
| 6500702 | S.flexneri 2a |
| 6500716 | S.flexneri 2a |
| 6500740 | S.flexneri 2a |
| 6500769 | S.flexneri 2a |
| 6500784 | S.sonnei |
| 6500791 | S.flexneri 2a |
| 6500896 | S.flexneri 2b |
| 6500908 | S.flexneri 1b |
| 6500913 | S.sonnei |
| 6500985 | S.sonnei |
| 6501040 | S.sonnei |
| 6501057 | S.flexneri 1a |
| 6501078 | S.flexneri 2b |
| 6501084 | Untyped |
| 6501091 | S.flexneri 2b |
| 6501103 | Untyped |
| 6501115 | Untyped |
| 6501126 | Untyped |
| 6501159 | Untyped |
| 6501209 | S.flexneri 2b |
| 6501227 | Untyped |
| 6501318 | S.flexneri 1b |
| 6501341 | S.sonnei |
| 6501352 | S.flexneri 1a |
| 6501365 | S.flexneri 2a |
| 6501376 | S.flexneri 2a |
| 6501424 | Untyped |
| 6501522 | S.sonnei |
| 6501645 | S.sonnei |
| 6501704 | S.flexneri 3a |
| 6501728 | S.flexneri 2a |
| 6501743 | S.sonnei |
| 6501755 | S.sonnei |
| 6501762 | S.flexneri 2a |
| 6501770 | S.flexneri 2a |
| 6501858 | S.flexneri 1b |
| 6501886 | S.flexneri 2a |
| 6501939 | S.flexneri 2a |
| 6501988 | Untyped |
| 6502003 | S.flexneri 3a |
| 6502015 | S.flexneri 2a |
| 6502098 | S.sonnei |
| 6502290 | S.sonnei |
