# Supplementary material for: Robust performance of culture, real-time PCR, and genomic approaches for shigella serotyping in a pediatric surveillance cohort
Source: PLoS Negl Trop Dis. 2026 Aug 26;20(8):e0014668. doi: 10.1371/journal.pntd.0014668 (PMC13614632; doi:10.1371/journal.pntd.0014668)
Supplement: Supplementary file 4 [file pntd.0014668.s004.docx]

**S4 Table. Serotype-specific sensitivity, specificity, and positive predictive value for all Shigella serotyping methods**

| **Serotype** | **Method** | **N evaluated** | **N with true serotype** | **TP** | **FP** | **FN** | **TN** | **Sensitivity (95% CI)** | **Specificity (95% CI)** | **PPV (95% CI)** |
| --- | --- | --- | --- | --- | --- | --- | --- | --- | --- | --- |
| 1a | Agglutination | 111 | 9 | 9 | 0 | 0 | 102 | 100.0% (70.1–100.0%) | 100.0% (96.4–100.0%) | 100.0% (70.1–100.0%) |
| 1a | Isolate-based real-time PCR | 107 | 8 | 2 | 1 | 6 | 98 | 25.0% (7.1–59.1%) | 99.0% (94.5–99.8%) | 66.7% (20.8–93.9%) |
| 1a | Stool-based real-time PCR | 111 | 9 | 2 | 1 | 7 | 101 | 22.2% (6.3–54.7%) | 99.0% (94.7–99.8%) | 66.7% (20.8–93.9%) |
| 1a | In-silico (ShigaPass) | 111 | 9 | 3 | 1 | 6 | 101 | 33.3% (12.1–64.6%) | 99.0% (94.7–99.8%) | 75.0% (30.1–95.4%) |
| 1b | Agglutination | 111 | 4 | 4 | 0 | 0 | 107 | 100.0% (51.0–100.0%) | 100.0% (96.5–100.0%) | 100.0% (51.0–100.0%) |
| 1b | Isolate-based real-time PCR | 107 | 3 | 3 | 6 | 0 | 98 | 100.0% (43.9–100.0%) | 94.2% (88.0–97.3%) | 33.3% (12.1–64.6%) |
| 1b | Stool-based real-time PCR | 111 | 4 | 2 | 6 | 2 | 101 | 50.0% (15.0–85.0%) | 94.4% (88.3–97.4%) | 25.0% (7.1–59.1%) |
| 1b | In-silico (ShigaPass) | 111 | 4 | 3 | 6 | 1 | 101 | 75.0% (30.1–95.4%) | 94.4% (88.3–97.4%) | 33.3% (12.1–64.6%) |
| 2a | Agglutination | 111 | 36 | 36 | 1 | 0 | 74 | 100.0% (90.4–100.0%) | 98.7% (92.8–99.8%) | 97.3% (86.2–99.5%) |
| 2a | Isolate-based real-time PCR | 107 | 34 | 34 | 0 | 0 | 73 | 100.0% (89.8–100.0%) | 100.0% (95.0–100.0%) | 100.0% (89.8–100.0%) |
| 2a | Stool-based real-time PCR | 111 | 36 | 34 | 0 | 2 | 75 | 94.4% (81.9–98.5%) | 100.0% (95.1–100.0%) | 100.0% (89.8–100.0%) |
| 2a | In-silico (ShigaPass) | 111 | 36 | 36 | 0 | 0 | 75 | 100.0% (90.4–100.0%) | 100.0% (95.1–100.0%) | 100.0% (90.4–100.0%) |
| 2b | Agglutination | 111 | 12 | 11 | 0 | 1 | 99 | 91.7% (64.6–98.5%) | 100.0% (96.3–100.0%) | 100.0% (74.1–100.0%) |
| 2b | Isolate-based real-time PCR | 107 | 12 | 12 | 0 | 0 | 95 | 100.0% (75.8–100.0%) | 100.0% (96.1–100.0%) | 100.0% (75.8–100.0%) |
| 2b | Stool-based real-time PCR | 111 | 12 | 11 | 0 | 1 | 99 | 91.7% (64.6–98.5%) | 100.0% (96.3–100.0%) | 100.0% (74.1–100.0%) |
| 2b | In-silico (ShigaPass) | 111 | 12 | 12 | 0 | 0 | 99 | 100.0% (75.8–100.0%) | 100.0% (96.3–100.0%) | 100.0% (75.8–100.0%) |
| 3a | Agglutination | 111 | 5 | 5 | 0 | 0 | 106 | 100.0% (56.6–100.0%) | 100.0% (96.5–100.0%) | 100.0% (56.6–100.0%) |
| 3a | Isolate-based real-time PCR | 107 | 5 | 5 | 0 | 0 | 102 | 100.0% (56.6–100.0%) | 100.0% (96.4–100.0%) | 100.0% (56.6–100.0%) |
| 3a | Stool-based real-time PCR | 111 | 5 | 5 | 1 | 0 | 105 | 100.0% (56.6–100.0%) | 99.1% (94.8–99.8%) | 83.3% (43.6–97.0%) |
| 3a | In-silico (ShigaPass) | 111 | 5 | 5 | 1 | 0 | 105 | 100.0% (56.6–100.0%) | 99.1% (94.8–99.8%) | 83.3% (43.6–97.0%) |
| 4a | Agglutination | 111 | 2 | 2 | 8 | 0 | 101 | 100.0% (34.2–100.0%) | 92.7% (86.2–96.2%) | 20.0% (5.7–51.0%) |
| 4a | Isolate-based real-time PCR | 107 | 2 | 2 | 0 | 0 | 105 | 100.0% (34.2–100.0%) | 100.0% (96.5–100.0%) | 100.0% (34.2–100.0%) |
| 4a | Stool-based real-time PCR | 111 | 2 | 1 | 0 | 1 | 109 | 50.0% (9.5–90.5%) | 100.0% (96.6–100.0%) | 100.0% (20.7–100.0%) |
| 4a | In-silico (ShigaPass) | 111 | 2 | 0 | 0 | 2 | 109 | 0.0% (0.0–65.8%) | 100.0% (96.6–100.0%) | N/A |
| Y | Agglutination | 111 | 3 | 2 | 0 | 1 | 108 | 66.7% (20.8–93.9%) | 100.0% (96.6–100.0%) | 100.0% (34.2–100.0%) |
| Y | Isolate-based real-time PCR | 107 | 3 | 0 | 0 | 3 | 104 | 0.0% (0.0–56.1%) | 100.0% (96.4–100.0%) | N/A |
| Y | Stool-based real-time PCR | 111 | 3 | 0 | 0 | 3 | 108 | 0.0% (0.0–56.1%) | 100.0% (96.6–100.0%) | N/A |
| Y | In-silico (ShigaPass) | 111 | 3 | 2 | 0 | 1 | 108 | 66.7% (20.8–93.9%) | 100.0% (96.6–100.0%) | 100.0% (34.2–100.0%) |
| Yv | Agglutination | 111 | 7 | 0 | 0 | 7 | 104 | 0.0% (0.0–35.4%) | 100.0% (96.4–100.0%) | N/A |
| Yv | Isolate-based real-time PCR | 107 | 7 | 0 | 0 | 7 | 100 | 0.0% (0.0–35.4%) | 100.0% (96.3–100.0%) | N/A |
| Yv | Stool-based real-time PCR | 111 | 7 | 0 | 0 | 7 | 104 | 0.0% (0.0–35.4%) | 100.0% (96.4–100.0%) | N/A |
| Yv | In-silico (ShigaPass) | 111 | 7 | 7 | 0 | 0 | 104 | 100.0% (64.6–100.0%) | 100.0% (96.4–100.0%) | 100.0% (64.6–100.0%) |
| sonnei | Agglutination | 111 | 33 | 33 | 0 | 0 | 78 | 100.0% (89.6–100.0%) | 100.0% (95.3–100.0%) | 100.0% (89.6–100.0%) |
| sonnei | Isolate-based real-time PCR | 107 | 33 | 33 | 0 | 0 | 74 | 100.0% (89.6–100.0%) | 100.0% (95.1–100.0%) | 100.0% (89.6–100.0%) |
| sonnei | Stool-based real-time PCR | 111 | 33 | 32 | 0 | 1 | 78 | 97.0% (84.7–99.5%) | 100.0% (95.3–100.0%) | 100.0% (89.3–100.0%) |
| sonnei | In-silico (ShigaPass) | 111 | 33 | 33 | 0 | 0 | 78 | 100.0% (89.6–100.0%) | 100.0% (95.3–100.0%) | 100.0% (89.6–100.0%) |

**N= number; TP= True positive; TN= True negative; FP= False positive; FN= False negative; SN= Sensitivity; SP= Specificity; PPV= Positive predictive value; CI= Confidence Interval**
