# Supplementary material for: Robust performance of culture, real-time PCR, and genomic approaches for shigella serotyping in a pediatric surveillance cohort
Source: PLoS Negl Trop Dis. 2026 Aug 26;20(8):e0014668. doi: 10.1371/journal.pntd.0014668 (PMC13614632; doi:10.1371/journal.pntd.0014668)
Supplement: Supplementary file 6 [file pntd.0014668.s006.docx]

March 3, 2025

Dr Godfred Menezes,
Dr. Stuart Blacksell
Dr. Shaden Kamhawi
Dr. Paul Brindley,

Editors
*PLoS Neglected Tropical Diseases*

Dear Dr. Menezes, Dr. Blacksell, Dr. Kamhawi and Dr. Brindley,

Thank your for reviewing the manuscript PNTD-D-26-00090 “***Robust Performance of Culture, qPCR, and Genomic Approaches for Shigella Serotyping in a Pediatric Surveillance Cohort”*** for consideration for publication in *PLoS Neglected Tropical* Diseases.

We have reviewed all comments and responded to each one individually. Additionally, we have made all changes effective in the manuscript draft, which has been resubmitted for your review. We feel the manuscript has improved since the original submission and hope to see the manuscript published in *PLoS Neglected Tropical Diseases.*

Sincerely,

**Margaret N. Kosek, MD**

Professor

Division of Infectious Diseases and International Health

School of Medicine

University of Virginia

**Reviewer 1**

**Comment A:** Throughout the manuscript, the authors use the term “quantitative PCR (qPCR),” but this is misleading because they did not actually quantify the bacteria. They used a real‑time PCR system, which is commonly employed for qPCR, but in this case it was not used for quantification.

**Response A:** the term quantitative PCR (q PCR) has been changed to real-time PCR throughout the manuscript.

**Comment B:** In the discussion section, the authors state that the agglutination method is subjective (Line 269). If that is the case, how did the authors ensure the accuracy and reproducibility of the agglutination method used in this study?

**Response B:** It is acknowledged that agglutination can be a subjective assay. However, in the present study, agglutination was ensured to be a reproducible procedure as described extensively in the microbiologic methods of the parent study (PMID 38532949). A note in the text has been made in Line 122-123.

Briefly from the reference manuscript: *“Quality control of antisera: All antisera will be verified using positive and negative controls at arrival, every month, and during technical training. Controls consist of a Shigella spp–positive control organism and an E coli–negative control organism, saline, and antisera in the following combinations: (a) Shigella spp plus saline, (b) saline plus antisera, (c) Shigella spp plus antisera, (d) E coli plus saline, and (e) E coli plus antisera (Supplementary Figure 3, Supplementary Tables 1 and 2). Agglutination with the listed antisera will be performed as per the polyvalent antisera and read after 1 minute.* Further quality control measures are described in the published reference manuscript.”

Horne B, Badji H, Bhuiyan MTR, Romaina Cachique L, Cornick J, Hotwani A, Juma J, Ochieng JB, Abdou M, Apondi E, Atlas HE, Awuor AO, Baker KS, Ceesay BE, Charles M, Cunliffe NA, Feutz E, Galagan SR, Guindo I, Hossain MJ, Iqbal J, Jallow F, Keita NY, Khanam F, Kotloff KL, Maiden V, Manzanares Villanueva K, Mito O, Mosharraf MP, Nkeze J, Ikumapayi UN, Paredes Olortegui M, Pavlinac PB, Pinedo Vasquez T, Qadri F, Qamar FN, Qureshi S, Rahman N, Sangare A, Sen S, Peñataro Yori P, Yousafzai MT, Ahmed D, Jere KC, Kosek MN, Omore R, Permala-Booth J, Secka O, Tennant SM. Microbiological Methods Used in the Enterics for Global Health *Shigella* Surveillance Study. Open Forum Infect Dis. 2024 Mar 25;11(Suppl 1):S25-S33. doi: 10.1093/ofid/ofad576. PMID: 38532949; PMCID: PMC10962722.

**Comment C:** Did the authors assess the reproducibility of each method? For instance, were PCR assays performed with technical and/or biological replicates, and if so, how consistent were the results of serotyping?

**Response C:** Agglutination, stool-based real-time PCR and isolate-based real-time PCR were run in duplicate for all samples for which a discrepant result was obtained. This has been added in line 173-174/

**Comment D:** Line 125 - In addition to the names of the target genes, please provide information on the sequences of primers used in these multiplex PCR assays. and the sizes of amplicons.

**Response D:** The list of primers and probes used for real-time based serotyping has been included as supplementary table 1.

**Comment E:** Line 135 - It is unclear whether the targets and primers used for the stool‑based PCR assay are the same as those used for the isolate‑based qPCR, or if they differ. Please clarify this point.

**Response E:** The targets (as well as the primers and probes) were the same for both isolate and stool-based PCR. This has been clarified in the methods section (Line 155-156).

**Comment F:** Line 161 (Supplementary Table 1) - In addition to genome completeness (coverage), please include information on the sequencing depth.

**Response F:** Coverage depth added to Supplementary Table 2 (previously labeled Supplementary Table 1)

**Comment G:** Table 4 appears to present sensitivity (true positive rate) as a performance indicator for each method; however, false positives have also been observed for some methods (as indicated by the red and pink cells in Figure 2. To provide a more comprehensive evaluation of performance, it would be advisable to include additional metrics such as specificity (true negative rate), precision, and negative predictive value, alongside sensitivity.

**Response G:** We thank the reviewer for pointing out the absence of SN, SP, PPV and NPV, and we agree that these metrics can be reported for individual serotypes, treating each as a binary classification (example: is this a serotype 1a: yes/no). However, conventional binary SN/SP/PPV/NPV metrics cannot be meaningfully aggregated into a single interpretable value without collapsing serotype specific estimates in a way that is not interpretable in this context. In this analysis, we are applying a classifier summary across a multi-class (in this case serotypes) problem. Our primary aim was to evaluate overall concordance with the WGS-adjudicated reference standard, for which percent accuracy with Wilson 95% CIs is the established and directly interpretable metric.

**Comment H:** Lines 213-215: Why weren’t whole‑genome sequencing or PCR assays performed on all available samples?

**Response H:** Whole genome sequencing was performed on all samples, however, genomes of good enough quality to predict the serotype were available for 111/114. Similarly, real-time PCR runs passing quality control were achieved from 110/114 samples.

**Comment I:** Line 220ff. Before introducing this paragraph, it would be better to present Figure 2 first. Once the interpretation of Figure 2 is understood, the content of this paragraph will become much clearer.

**Response I:** Figure 2 has been introduced in the second paragraph of the results section.

**Comment J:** Figure 2 -I assume that the numbers in these matrices represent the number of isolates, but please explicitly state that this is the case. Please also provide an explanation of the color scale used in the matrices (e.g., red, pink, gray). Additionally, I couldn’t understand part of the legend “Comparison of the four serotyping tools … reveals that both agglutination and the in-silico tool assign correctly the same proportion of samples.” Please elaborate.

**Comment K:** Line 268 - sensitivity sensitivity --> sensitivity

**Response K:** Duplicate word has been deleted (line 307).

Comment L: Line 320 - coverage --> depth

**Response L:** The term coverage has been updated to coverage depth.

**Comment M:** Lines 319-322. Are there any evidences and/or literature that support this claim? The literature (35) is about SPAdes itself. I'm not sure why the selection of sequencing depth and the contig‑length threshold have such a strong impact on Shigella serotype identification.

**Response M:** We thank the reviewer for bringing this to our attention. We have altered the sentences to make it clear and correct. Line 391-396: “*ShigaPass requires the complete genome assembly as input, including contigs below standard-length thresholds. Our default assembly pipeline applied a 500 nt minimum contig cutoff; however, for one isolate this excluded a contig carrying a fragment of ipaH, reducing the gene representation below the minimum kmer threshold required for ShigaPass identification. Lowering the contig cutoff to include contigs below 500 nt for this isolate recovered the ipaH-containing contig and enabled successful species assignment.*”

**Reviewer 2**

**Comment A:** Clarification of Sample Size Fluctuations: The authors frequently change denominators when describing different methods (e.g., 114, 111, 110, 107, 98). While the manuscript explains these variations as results of experimental failure or data unavailability, I recommend including a Flowchart. This should clearly illustrate the specific number of samples included in each comparative analysis, starting from the initial 114 isolates, to enhance transparency.

**Response A:** A flow-chart has been created and included as Supplementary Figure 1, as well as cited in the text (Line 236)

**Comment B:** Verification of Percentage Calculations: * In line 216, 77/110 is cited as 70.0%, which is correct. However, in line 217, 78/111 is approximately 70.27%; I suggest consistently rounding to one decimal place throughout the manuscript.

**Response B:** All percentages have been rounded to one decimal place. All calculations have been revised for rounding consistency.

**Comment C:** In line 245, should the accuracy for 102/111 be 91.9% or 91.89%? Please verify the rounding rules for all percentages to ensure global consistency.

**Response C:** 102/111 is presented as 91.9%, which is 91.89% rounded to one decimal place.

**Comment D:** Establishment of the Gold Standard: Line 244 mentions using "adjudicated manual sequence analysis" as the gold standard. The authors need to provide a more detailed definition of the "adjudication" process. Since the study identified nonsense mutations in certain genes (e.g., oac and Petn-transferase) that lead to discrepancies between genotype and phenotype, the authors should clarify whether the gold standard is based on "functional gene prediction" or "whole-genome phylogenetic relationships."

**Response D:** We have described the adjudicated manual sequence analysis in more detail in the Materials and Methods section. The full paragraph now reads:

Line 190-223: *“Manual genome assessment for serotype adjudication was based on presence/absence of S. flexneri O-antigen modification genes and assessment of gene integrity. Detection of O-antigen modification genes and identification of premature stop codon mutations indicative of gene inactivation were performed using the Map to Reference function in Geneious Prime (v2025.1.2; Biomatters, Ltd., Auckland, New Zealand). Illumina paired-end reads >150 nt generated from an individual S. flexneri strain were mapped to 8 O-antigen biosynthesis and modification regions: wba (AE005674 (SF2096-SF2104)), gtrABI (CP130063 (QS169_18520-QS169_18530)), oac1b (JN377795), gtrABII (AE005674 (SF0305- SF0307)), gtrX (L05001), oac (AF547987), gtrIV (NC_022749 (V416_gp25)), and opt (KC020049). All S. flexneri serotypes 1–5, X and Y should have sequence reads that map across the wba locus, which encodes S. flexneri O-antigen backbone biosynthesis enzymes. Both S. flexneri 1a and S. flexneri 1b strains should have sequence reads that map to the gtrABI locus, and S. flexneri 1b should have sequence reads that map to oac1b. Both S. flexneri 2a and S. flexneri 2b strains should have sequence reads that map to the gtrABII and S. flexneri 2b should have sequence reads that map to gtrX. Both S. flexneri 3a and S. flexneri 3b strains should have sequence reads that map to oac, and S. flexneri 3a strains should have sequence reads that map to gtrX. S. flexneri 4a, S. flexneri 4av and S. flexneri 4b strains should have sequence reads that map to gtrIV, S. flexneri 4av strains should have sequence reads that map to opt, and S. flexneri 4b strains should have sequence reads that map to oac. S. flexneri Y strains have reads that only map to the wba locus and S. flexneri Yv strains should have sequence reads that map to opt. Genomic assemblies with ≥5× coverage across O-antigen modification genes were considered sufficient for two assessments: detection of gene presence or absence, and identification of premature stop codons arising from frameshift or nonsense mutations. Stop codon calls were accepted only when the causative mutation was present in all mapped reads at that position, confirming a true chromosomal mutation rather than a sequencing artefact.”*

**Comment E:** Handling of Serotypes Y and Yv: qPCR is unable to detect Y and Yv (Line 224). The exclusion (Accuracy 91.8%) versus inclusion (Accuracy 84.1%) of these "non-typable" results leads to significant differences in accuracy metrics. In the conclusion, the authors should more objectively emphasize the limitations of qPCR in regions where these specific serotypes are prevalent.

**Response E:** We have updated the discussion to emphasize the limitation qPCR-based serotyping in potential geographic areas where the Y and Yv isolates are highly prevalent (line 354-357). Text now reads: “*It is limited, however, by its inability to detect Y and Yv serotypes. Although these serotypes have relatively low prevalence in most settings, the application of qPCR-based serotyping would be restricted in geographic areas where they are more prevalent”*

**Reviewer 3**

**Comment A:** The analysis is based on 107–114 isolates derived from a single study site in Iquitos, Peru. The relatively small number of isolates reduces statistical power, especially for less frequent serotypes.

**Response A:** Although the present analysis is based on only 114 isolates, it is derived from an observational parent study conducted in 7 sites (including Peru). The methodological aspects of the parent study have been previously published (PMID 38532963), as well as cited in the present text line (102). Briefly: The parent study was designed to detect a “a *minimum number of 65 culture-confirmed Shigella cases. This would ensure that at least 5 isolates from the 4 leading species/subserotypes (S. sonnei, S. flexneri 2a, S. flexneri 3a, and S. flexneri 6) at each site and to achieve appropriate precision (width less <3%) for a 2-sided 95% confidence interval around the observed proportion of children enrolled with culture-positive Shigella.* *We assumed that 4.7% of diarrhea cases in EFGH would be Shigella spp. culture positive, requiring that we enroll 1400 participants presenting to health facilities with diarrhea over the 24-month period (∼58 participants per month)”.*

Atlas HE, Conteh B, Islam MT, Jere KC, Omore R, Sanogo D, Schiaffino F, Yousafzai MT, Ahmed N, Awuor AO, Badji H, Cornick J, Feutz E, Galagan SR, Haidara FC, Horne B, Hossen MI, Hotwani A, Houpt ER, Jallow AF, Karim M, Keita AM, Keita Y, Khanam F, Liu J, Malemia T, Manneh A, McGrath CJ, Nasrin D, Ndalama M, Ochieng JB, Ogwel B, Paredes Olortegui M, Zegarra Paredes LF, Pinedo Vasquez T, Platts-Mills JA, Qudrat-E-Khuda S, Qureshi S, Hasan Rajib MN, Rogawski McQuade ET, Sultana S, Tennant SM, Tickell KD, Witte D, Peñataro Yori P, Cunliffe NA, Hossain MJ, Kosek MN, Kotloff KL, Qadri F, Qamar FN, Tapia MD, Pavlinac PB. Diarrhea Case Surveillance in the Enterics for Global Health *Shigella* Surveillance Study: Epidemiologic Methods. Open Forum Infect Dis. 2024 Mar 25;11(Suppl 1):S6-S16. doi: 10.1093/ofid/ofad664. PMID: 38532963; PMCID: PMC10962728.

**Comment B**: No Shigella dysenteriae or Shigella boydii isolates were detected, limiting conclusions to mainly S. flexneri and S. sonnei.

**Response B:** No conclusions or inferences were made for *Shigella dysenteriae* or *Shigella boydii*.

**Comment C:** The geographic restriction reduces external validity.

**Response C:** The geographic restriction of this analysis was already noted in the discussion. However, it has been highlighted in Line 422-423. It now reads: “*The present study is limited by the restricted number of serotypes found in Loreto, Peru, as well as the geographic restriction of the population sampled. Thus, serotyping performance of other serotypes not found in this geographic region could not be determined. That said, the serotypes found in this population are representative of the most frequently identified in large scale epidemiologic studies (39).”*

**Comment D:** The manuscript designates expert WGS-based analysis using Geneious Prime as the definitive reference method. Criteria for expert adjudication are insufficiently detailed. Inter-rater reliability and reproducibility are not addressed. The process may introduce subjective bias.

**Response D:** We have described the adjudicated manual sequence analysis in more detail in the Materials and Methods section. Please see reviewer 2, response D.

**Comment E:** Accuracy is calculated mainly using Wilson confidence intervals, and concordance percentages are reported**.** Limitations include:

- Absence of sensitivity, specificity, PPV, and NPV.
- No formal agreement analysis (e.g., Cohen’s kappa).
- No statistical comparison between methods.
- Provide justification for exclusion criteria and perform sensitivity analyses to demonstrate robustness of results.

**Response E:**

We thank the reviewer for pointing out the absence of SN, SP, PPV and NPV, and we agree that these metrics can be reported for individual serotypes, treating each as a binary classification (example: is this a serotype 1a: yes/no). However, conventional binary SN/SP/PPV/NPV metrics cannot be meaningfully aggregated into a single interpretable value without collapsing serotype specific estimates in a way that is not interpretable in this context. In this analysis, we are applying a classifier summary across a multi-class (in this case serotypes) problem. Our primary aim was to evaluate overall concordance with the WGS-adjudicated reference standard, for which percent accuracy with Wilson 95% CIs is the established and directly interpretable metric.

Cohen’s Kappa is statistic used to compare the classification of two independent fallible raters. However, in this case, the WGS adjudicated standard is defined as the gold standard or truth, not an equal rater and thus making the interpretation of Cohen’s Kappa limited.

We have performed McNemar's test for paired proportions and, as expected, determined that there is no statistically significant difference in the overall accuracy between methods. However, we have not included this statistic in the manuscript given that this study was designed to evaluate concordance with the WGS adjudicated standard, not to establish superiority of one method over another. Evidence for equivalence would be misleading given that misclassification errors are “multi-class”. In other words, the test treats the misclassification error of Y vs. 4a the same as 2a vs 1b, yet, these are the result of different underlying biological processes.

Of the 114 *Shigella* isolates available, 7 were excluded from the primary four-method comparison because complete results were not available across all methods: WGS was not successfully completed for 3 isolates, and isolate-based qPCR was not performed on 4 isolates, due to technical failures unrelated to the isolate's serotype. These exclusions were not selective with respect to any particular serotype or method outcome, and their small number (6.1%) is unlikely to introduce bias. Additionally, we performed this analysis analyzing each pairwise method using all available isolate pairs, as is shown in the results section of the manuscript (lines 288-302).

**Comment F:** The manuscript acknowledges that qPCR fails to detect Y/Yv serotypes and nonsense mutations. Similarly, ShigaPass fails under low coverage and cannot detect loss-of-function mutations. However, these limitations are not quantitatively assessed.

**Response F:** The results presented in the manuscript are the quantitative assessment of the limitations of these serotyping assays.

**Comment G:** Include approximate cost, infrastructure, and personnel requirements for each method, especially for LMIC settings.

**Response G:** The three methods differ in cost, infrastructure and analytic complexity as suggested by the reviewer. The costs are clearly less for serotyping as compared with PCR, which are considerably less than sequencing. Individual costs to laboratories differ greatly, and depending on existing infrastructure, the exact pricing is difficult to predict in absolute terms in different contexts. It should be noted that laboratories in LMIC are highly heterogeneous in laboratory capacity and infrastructure. After the COVID-19 pandemic, many laboratories gained capacity in sequencing that would make genomic typing feasible.

**Final comment:** the reviewer proved a bullet point list of items to improve (see list below), that have already been addressed in Comments A – G).

- Improve transparency and reproducibility of the WGS adjudication process.
- Expand statistical analyses and agreement metrics.
- Address sample size and geographic limitations.
- Provide more rigorous treatment of negative and non-typed samples.
- Quantify methodological limitations and practical feasibility.
- Strengthen discussion on generalizability beyond vaccine serotypes.
- Addressing these points will substantially enhance the scientific rigor and practical relevance of the study.
