# Supplementary material for: Robust performance of culture, real-time PCR, and genomic approaches for shigella serotyping in a pediatric surveillance cohort
Source: PLoS Negl Trop Dis. 2026 Aug 26;20(8):e0014668. doi: 10.1371/journal.pntd.0014668 (PMC13614632; doi:10.1371/journal.pntd.0014668)
Supplement: Supplementary file 7 [file pntd.0014668.s007.docx]

May 20, 2026

Dr Godfred Menezes,
Dr. Stuart Blacksell
Dr. Shaden Kamhawi
Dr. Paul Brindley,

Editors
*PLoS Neglected Tropical Diseases*

Dear Dr. Menezes, Dr. Blacksell, Dr. Kamhawi and Dr. Brindley,

Thank you for the second review of the manuscript PNTD-D-26-00090 “***Robust Performance of Culture, qPCR, and Genomic Approaches for Shigella Serotyping in a Pediatric Surveillance Cohort”*** for consideration for publication in *PLoS Neglected Tropical* Diseases.

We have reviewed all comments and responded to each one individually. Additionally, we have made all changes effective in the manuscript draft, which has been resubmitted for your review. We feel the manuscript has improved since the original submission and hope to see the manuscript published in *PLoS Neglected Tropical Diseases.*

Sincerely,

**Margaret N. Kosek, MD**

Professor

Division of Infectious Diseases and International Health

School of Medicine

University of Virginia

**____________________________________________________________________________________**

**Reviewer #1**

**Comment A:** The authors did not respond to my previous comment on Figure 2. Previously, I commented “I assume that the numbers in these matrices represent the number of isolates, but please explicitly state that this is the case. Please also provide an explanation of the color scale used in the matrices (e.g., red, pink, gray). Additionally, I couldn’t understand part of the legend “Comparison of the four serotyping tools … reveals that both agglutination and the in-silico tool assign correctly the same proportion of samples.” Please elaborate.”
**Response A: The legend of Figure 2 has been updated to better clarify its content. It now reads: *“****Each panel shows a serotype concordance matrix in which rows represent the assigned serotype, columns represent the definitive serotype, and cell values indicate the number of isolates. Cells colored in pink or red indicate misclassifications, with color intensity proportional to the number of misclassified isolates. Gray cells indicate isolates that were undetected or non-typable by the method.”*

**Comment B:** Regarding the performance metrics, I largely agree with the authors' response. However, I disagree with the use of the word "accuracy" (for example, in Table 4). In this case, the word "sensitivity" would be more appropriate. Also, I agree that evaluating true negatives may be difficult without collapsing serotype specific results, but evaluating false positives should be possible, and therefore the authors can calculate PPV (similar to how the authors calculated the sensitivity).
**Response B:** We thank the reviewer for this comment and appreciate the continued engagement on this matter. We respectfully maintain the use of "***accuracy***" as the appropriate metric for this analysis and would like to clarify why.

When performing serotyping, we assign each isolate to one of many different possible serotypes. We do not assign a binary outcome (presence/absence). Thus, in this analysis, accuracy is defined as the proportion of correct serotype assignments out of all the isolates that were evaluated. If we applied sensitivity, we would need to collapse all possible assignments into a binary classification (such as vaccine relevant vs. not relevant). In theory, we could do this and calculate it, yet it does not reflect how each serotyping classification method operates. With regards to Table 4, we have already restricted the denominator to isolates confirmed as vaccine relevant serotypes by the manual adjudication (“gold standard”), thus, by design, this table does not contain true negatives or false positives. In other words, all isolates in the table are either true positive or false negatives. Adding a PPV to Table 4 would thus require restructuring the analysis to include all isolates regardless of serotype.

**That said, we agree that it would be of interest to the readership to know these metrics and thus have added a supplementary table in which the SN, SP and PPV of each serotype and method. It is now named Supplementary Table 4. A paragraph in the methods section has also been included.**

**Comment C:** The authors have responded appropriately to most of my comments, but I believe some of my comments have not been addressed and therefore require further clarification (see my comments for “Results”).
**Response C:** See response to comment A and B lines above.

**Reviewer #4**

**Comment D:** This study provides a robust Shigella serotyping scheme/protocol. The revised version also fully and reasonably addresses the concerns raised by the previous reviewers. Therefore, I support the publication of this article.
**Response D:** We thank the reviewer.

**Reviewer #5**

**Comment E:** The methods are nicely detailed and responsive to the prior critiques.
**Response E:** No changes to the methods section have been made since the last submission.

**Comment F:** The analysis presented is clear and concise
**Response F:** We thank the reviewer.

**Comment G:** Overall this is a valuable addition to the field that compares the utility of 4 different methods to assess Shigella serotyping, something that is somewhat of a logistical challenge with traditional methods. It is anticipated that this study will provide standardized methodology that can be exploited in vaccine trials.
**Response G:** We thank the reviewer.
