# Supplementary material for: Robust performance of culture, real-time PCR, and genomic approaches for shigella serotyping in a pediatric surveillance cohort
Source: PLoS Negl Trop Dis. 2026 Aug 26;20(8):e0014668. doi: 10.1371/journal.pntd.0014668 (PMC13614632; doi:10.1371/journal.pntd.0014668)
Supplement: Supplementary file 8 [file pntd.0014668.s005.docx]

**S1 Fig .** Flow chart showing sample selection and inclusion in the study.
