# Supplementary material for: Robust performance of culture, real-time PCR, and genomic approaches for shigella serotyping in a pediatric surveillance cohort
Source: PLoS Negl Trop Dis. 2026 Aug 26;20(8):e0014668. doi: 10.1371/journal.pntd.0014668 (PMC13614632; doi:10.1371/journal.pntd.0014668)
Supplement: Peer review history

# Peer review: Decision Letter 0

**DOI:** 10.1371/journal.pntd.0014668.r001

**Authors:** Stuart D. Blacksell, Godfred Menezes

27 Feb 2026

Robust Performance of Culture, qPCR, and Genomic Approaches for Shigella Serotyping in a Pediatric Surveillance Cohort

PLOS Neglected Tropical Diseases

Dear Dr. Kosek,

Thank you for submitting your manuscript to PLOS Neglected Tropical Diseases. After careful consideration, we feel that it has merit but does not fully meet PLOS Neglected Tropical Diseases's publication criteria as it currently stands. Therefore, we invite you to submit a revised version of the manuscript that addresses the points raised during the review process.

Please submit your revised manuscript within by Apr 28 2026 11:59PM. If you will need more time than this to complete your revisions, please reply to this message or contact the journal office at plosntds@plos.org. When you're ready to submit your revision, log on to https://www.editorialmanager.com/pntd/ and select the 'Submissions Needing Revision' folder to locate your manuscript file.

Please include the following items when submitting your revised manuscript:

* A letter that responds to each point raised by the editor and reviewer(s). You should upload this letter as a separate file labeled 'Response to Reviewers'. This file does not need to include responses to any formatting updates and technical items listed in the 'Journal Requirements' section below.

* A marked-up copy of your manuscript that highlights changes made to the original version. You should upload this as a separate file labeled 'Revised Manuscript with Track Changes'.

* An unmarked version of your revised paper without tracked changes. You should upload this as a separate file labeled 'Manuscript'.

If you would like to make changes to your financial disclosure, competing interests statement, or data availability statement, please make these updates within the submission form at the time of resubmission. Guidelines for resubmitting your figure files are available below the reviewer comments at the end of this letter.

We look forward to receiving your revised manuscript.

Kind regards,

Godfred Menezes

Academic Editor

PLOS Neglected Tropical Diseases

Stuart Blacksell

Section Editor

PLOS Neglected Tropical Diseases

Shaden Kamhawi

co-Editor-in-Chief

PLOS Neglected Tropical Diseases

orcid.org/0000-0003-4304-636XX

Paul Brindley

co-Editor-in-Chief

PLOS Neglected Tropical Diseases

orcid.org/0000-0003-1765-0002

**Journal Requirements:**

1) Please provide an Author Summary. This should appear in your manuscript between the Abstract (if applicable) and the Introduction, and should be 150-200 words long. The aim should be to make your findings accessible to a wide audience that includes both scientists and non-scientists. Sample summaries can be found on our website under Submission Guidelines:

https://journals.plos.org/plosntds/s/submission-guidelines#loc-parts-of-a-submission

2) Please upload all main figures as separate Figure files in .tif or .eps format. For more information about how to convert and format your figure files please see our guidelines:

https://journals.plos.org/plosntds/s/figures

3) We have noticed that you have uploaded Supporting Information files, but you have not included a list of legends. Please add a full list of legends for your Supporting Information files after the references list.

4) Please amend your detailed Financial Disclosure statement. This is published with the article. It must therefore be completed in full sentences and contain the exact wording you wish to be published.

State what role the funders took in the study. If the funders had no role in your study, please state: "The funders had no role in study design, data collection and analysis, decision to publish, or preparation of the manuscript.".

If you did not receive any funding for this study, please simply state: u201cThe authors received no specific funding for this work.u201d

**Reviewers' Comments:**

Reviewer's Responses to Questions

**Key Review Criteria Required for Acceptance?**

As you describe the new analyses required for acceptance, please consider the following:

**Methods**

-Are the objectives of the study clearly articulated with a clear testable hypothesis stated?

-Is the study design appropriate to address the stated objectives?

-Is the population clearly described and appropriate for the hypothesis being tested?

-Is the sample size sufficient to ensure adequate power to address the hypothesis being tested?

-Were correct statistical analysis used to support conclusions?

-Are there concerns about ethical or regulatory requirements being met?

Reviewer #1: The manuscript is clearly written, and the goal, hypotheses and design of the study are clear. I don't think there is any ethical and regulatory problem in the study.

Throughout the manuscript, the authors use the term “quantitative PCR (qPCR),” but this is misleading because they did not actually quantify the bacteria. They used a real‑time PCR system, which is commonly employed for qPCR, but in this case it was not used for quantification.

In the discussion section, the authors state that the agglutination method is subjective (Line 269). If that is the case, how did the authors ensure the accuracy and reproducibility of the agglutination method used in this study?

Did the authors assess the reproducibility of each method? For instance, were PCR assays performed with technical and/or biological replicates, and if so, how consistent were the results of serotyping?

Line 125

In addition to the names of the target genes, please provide information on the sequences of primers used in these multiplex PCR assays. and the sizes of amplicons.

Line 135

It is unclear whether the targets and primers used for the stool‑based PCR assay are the same as those used for the isolate‑based qPCR, or if they differ. Please clarify this point.

Line 161 (Supplementary Table 1)

In addition to genome completeness (coverage), please include information on the sequencing depth.

Reviewer #2: (No Response)

Reviewer #3: (No Response)

**********

**Results**

-Does the analysis presented match the analysis plan?

-Are the results clearly and completely presented?

-Are the figures (Tables, Images) of sufficient quality for clarity?

Reviewer #1: The results are reported in an easy-to-understand manner in accordance with the research plan and methodology presented in the manuscript. The figures are generally well prepared and clearly presented.

Table 4 appears to present sensitivity (true positive rate) as a performance indicator for each method; however, false positives have also been observed for some methods (as indicated by the red and pink cells in Figure 2. To provide a more comprehensive evaluation of performance, it would be advisable to include additional metrics such as specificity (true negative rate), precision, and negative predictive value, alongside sensitivity.

Lines 213-215

Why weren’t whole‑genome sequencing or PCR assays performed on all available samples?

Line 220ff.

Before introducing this paragraph, it would be better to present Figure 2 first. Once the interpretation of Figure 2 is understood, the content of this paragraph will become much clearer.

Figure 2

I assume that the numbers in these matrices represent the number of isolates, but please explicitly state that this is the case. Please also provide an explanation of the color scale used in the matrices (e.g., red, pink, gray). Additionally, I couldn’t understand part of the legend “Comparison of the four serotyping tools … reveals that both agglutination and the in-silico tool assign correctly the same proportion of samples.” Please elaborate.

Reviewer #2: (No Response)

Reviewer #3: (No Response)

**********

**Conclusions**

-Are the conclusions supported by the data presented?

-Are the limitations of analysis clearly described?

-Do the authors discuss how these data can be helpful to advance our understanding of the topic under study?

-Is public health relevance addressed?

Reviewer #1: The significance and public health relevance of the study are clearly explained. I was also able to understand how the data provided in this study are important for the prevention of shigellosis for the advancement of the topic under study. The study also appropriately discusses its limitations, emphasizing that the noted issues stem from the methods evaluated rather than from the study itself.

Reviewer #2: (No Response)

Reviewer #3: (No Response)

**********

**Editorial and Data Presentation Modifications?**

Use this section for editorial suggestions as well as relatively minor modifications of existing data that would enhance clarity. If the only modifications needed are minor and/or editorial, you may wish to recommend “Minor Revision” or “Accept”.

Reviewer #1: Line 268

sensitivity sensitivity --> sensitivity

Line 320

coverage --> depth

Lines 319-322

Are there any evidences and/or literature that support this claim? The literature (35) is about SPAdes itself. I'm not sure why the selection of sequencing depth and the contig‑length threshold have such a strong impact on Shigella serotype identification.

Reviewer #2: (No Response)

Reviewer #3: (No Response)

**********

**Summary and General Comments**

Use this section to provide overall comments, discuss strengths/weaknesses of the study, novelty, significance, general execution and scholarship. You may also include additional comments for the author, including concerns about dual publication, research ethics, or publication ethics. If requesting major revision, please articulate the new experiments that are needed.

Reviewer #1: The manuscript is clearly written, and the goal of the study is clear. The methods used in the experiment are mostly reasonable. This research is believed to be meaningful from a perspective of public health.

Reviewer #2: This manuscript provides a comprehensive comparative analysis of four Shigella serotyping methods: isolate-based agglutination, isolate-based qPCR, stool-based qPCR, and in silico genomic prediction. Given the diversity of Shigella serotypes and their critical role in vaccine development, this research holds significant clinical and public health value. However, I have a few questions regarding the rigor of data presentation and the derivation of certain conclusions.

1. Data Consistency & Accuracy

Clarification of Sample Size Fluctuations: The authors frequently change denominators when describing different methods (e.g., 114, 111, 110, 107, 98). While the manuscript explains these variations as results of experimental failure or data unavailability, I recommend including a Flowchart. This should clearly illustrate the specific number of samples included in each comparative analysis, starting from the initial 114 isolates, to enhance transparency.

Verification of Percentage Calculations: * In line 216, 77/110 is cited as 70.0%, which is correct. However, in line 217, 78/111 is approximately 70.27%; I suggest consistently rounding to one decimal place throughout the manuscript.

In line 245, should the accuracy for 102/111 be 91.9% or 91.89%? Please verify the rounding rules for all percentages to ensure global consistency.

2. Definition of the "Gold Standard"

Establishment of the Gold Standard: Line 244 mentions using "adjudicated manual sequence analysis" as the gold standard. The authors need to provide a more detailed definition of the "adjudication" process. Since the study identified nonsense mutations in certain genes (e.g., oac and Petn-transferase) that lead to discrepancies between genotype and phenotype, the authors should clarify whether the gold standard is based on "functional gene prediction" or "whole-genome phylogenetic relationships."

Handling of Serotypes Y and Yv: qPCR is unable to detect Y and Yv (Line 224). The exclusion (Accuracy 91.8%) versus inclusion (Accuracy 84.1%) of these "non-typable" results leads to significant differences in accuracy metrics. In the conclusion, the authors should more objectively emphasize the limitations of qPCR in regions where these specific serotypes are prevalent.

Reviewer #3: The study evaluates agglutination, isolate-based qPCR, stool-based qPCR, and WGS-based serotyping in a pediatric cohort from Peru and reports high accuracy for vaccine-relevant serotypes.

The topic is timely and relevant, particularly for vaccine trials and surveillance programs. The manuscript is generally well written and methodologically sound. However, several aspects limit its broader applicability and scientific rigor, warranting major revision.

Limited Sample Size and Representativeness

The analysis is based on 107–114 isolates derived from a single study site in Iquitos, Peru

The relatively small number of isolates reduces statistical power, especially for less frequent serotypes.

No Shigella dysenteriae or Shigella boydii isolates were detected, limiting conclusions to mainly S. flexneri and S. sonnei.

The geographic restriction reduces external validity.

The manuscript designates expert WGS-based analysis using Geneious Prime as the definitive reference method

However:

Criteria for expert adjudication are insufficiently detailed.

Inter-rater reliability and reproducibility are not addressed.

The process may introduce subjective bias.

Statistical Analysis and Performance Metrics

Accuracy is calculated mainly using Wilson confidence intervals, and concordance percentages are reported

Limitations include:

Absence of sensitivity, specificity, PPV, and NPV.

No formal agreement analysis (e.g., Cohen’s kappa).

No statistical comparison between methods.

Provide justification for exclusion criteria and perform sensitivity analyses to demonstrate robustness of results.

The manuscript acknowledges that qPCR fails to detect Y/Yv serotypes and nonsense mutations

Similarly, ShigaPass fails under low coverage and cannot detect loss-of-function mutations

However, these limitations are not quantitatively assessed.

Include approximate cost, infrastructure, and personnel requirements for each method, especially for LMIC settings.

Summary Recommendation to Authors

This manuscript addresses an important problem and provides valuable comparative data. However, before publication, the following major issues must be addressed:

Improve transparency and reproducibility of the WGS adjudication process.

Expand statistical analyses and agreement metrics.

Address sample size and geographic limitations.

Provide more rigorous treatment of negative and non-typed samples.

Quantify methodological limitations and practical feasibility.

Strengthen discussion on generalizability beyond vaccine serotypes.

Addressing these points will substantially enhance the scientific rigor and practical relevance of the study.

**********

PLOS authors have the option to publish the peer review history of their article (what does this mean?). If published, this will include your full peer review and any attached files.

If you choose “no”, your identity will remain anonymous but your review may still be made public.

**Do you want your identity to be public for this peer review?**  For information about this choice, including consent withdrawal, please see our Privacy Policy.

Reviewer #1: No

Reviewer #2: No

Reviewer #3: No

[NOTE: If reviewer comments were submitted as an attachment file, they will be attached to this email and accessible via the submission site. Please log into your account, locate the manuscript record, and check for the action link "View Attachments". If this link does not appear, there are no attachment files.]

**Figure resubmission:**

**Reproducibility:**

To enhance the reproducibility of your results, we recommend that authors of applicable studies deposit laboratory protocols in protocols.io, where a protocol can be assigned its own identifier (DOI) such that it can be cited independently in the future. Additionally, PLOS ONE offers an option to publish peer-reviewed clinical study protocols. Read more information on sharing protocols at https://plos.org/protocols?utm_medium=editorial-email&utm_source=authorletters&utm_campaign=protocols

# Author comment: Author response to Decision Letter 1

**DOI:** 10.1371/journal.pntd.0014668.r002

10 Mar 2026

Attachment Submitted filename: ShigellaSerotypingComparison_ResponseReviewers_submit.docx

# Peer review: Decision Letter 1

**DOI:** 10.1371/journal.pntd.0014668.r003

**Authors:** Stuart D. Blacksell, Godfred Menezes

12 May 2026

Response to Reviewers Revised Manuscript with Track Changes Manuscript

Godfred Menezes

Academic Editor

PLOS Neglected Tropical Diseases

Shaden Kamhawi

co-Editor-in-Chief

PLOS Neglected Tropical Diseases

orcid.org/0000-0003-4304-636XX

Paul Brindley

co-Editor-in-Chief

PLOS Neglected Tropical Diseases

orcid.org/0000-0003-1765-0002

**Reviewers' comments:**

**Key Review Criteria Required for Acceptance?**

As you describe the new analyses required for acceptance, please consider the following:

**Methods**

-Are the objectives of the study clearly articulated with a clear testable hypothesis stated?

-Is the study design appropriate to address the stated objectives?

-Is the population clearly described and appropriate for the hypothesis being tested?

-Is the sample size sufficient to ensure adequate power to address the hypothesis being tested?

-Were correct statistical analysis used to support conclusions?

-Are there concerns about ethical or regulatory requirements being met?

Reviewer #1: (No Response)

Reviewer #4: (No Response)

Reviewer #5: The methods are nicely detailed and responsive to the prior critiques.

**********

**Results**

-Does the analysis presented match the analysis plan?

-Are the results clearly and completely presented?

-Are the figures (Tables, Images) of sufficient quality for clarity?

Reviewer #1: The authors have responded appropriately to most of my comments, but I believe the following points require further clarification.

The authors did not respond to my previous comment on Figure 2. Previously, I commented “I assume that the numbers in these matrices represent the number of isolates, but please explicitly state that this is the case. Please also provide an explanation of the color scale used in the matrices (e.g., red, pink, gray). Additionally, I couldn’t understand part of the legend “Comparison of the four serotyping tools … reveals that both agglutination and the in-silico tool assign correctly the same proportion of samples.” Please elaborate.”

Regarding the performance metrics, I largely agree with the authors' response. However, I disagree with the use of the word "accuracy" (for example, in Table 4). In this case, the word "sensitivity" would be more appropriate. Also, I agree that evaluating true negatives may be difficult without collapsing serotype specific results, but evaluating false positives should be possible, and therefore the authors can calculate PPV (similar to how the authors calculated the sensitivity).

Reviewer #4: (No Response)

Reviewer #5: The analysis presented is clear and concise

**********

**Conclusions**

-Are the conclusions supported by the data presented?

-Are the limitations of analysis clearly described?

-Do the authors discuss how these data can be helpful to advance our understanding of the topic under study?

-Is public health relevance addressed?

Reviewer #1: (No Response)

Reviewer #4: (No Response)

Reviewer #5: Overall this is a valuable addition to the field that compares the utility of 4 different methods to assess Shigella serotyping, something that is somewhat of a logistical challenge with traditional methods. It is anticipated that this study will provide standardized methodology that can be exploited in vaccine trials.

**********

**Editorial and Data Presentation Modifications?**

Use this section for editorial suggestions as well as relatively minor modifications of existing data that would enhance clarity. If the only modifications needed are minor and/or editorial, you may wish to recommend “Minor Revision” or “Accept”.

Reviewer #1: (No Response)

Reviewer #4: (No Response)

Reviewer #5: (No Response)

**********

**Summary and General Comments**

Use this section to provide overall comments, discuss strengths/weaknesses of the study, novelty, significance, general execution and scholarship. You may also include additional comments for the author, including concerns about dual publication, research ethics, or publication ethics. If requesting major revision, please articulate the new experiments that are needed.

Reviewer #1: The authors have responded appropriately to most of my comments, but I believe some of my comments have not been addressed and therefore require further clarification (see my comments for “Results”).

Reviewer #4: This study provides a robust Shigella serotyping scheme/protocol. The revised version also fully and reasonably addresses the concerns raised by the previous reviewers. Therefore, I support the publication of this article.

Reviewer #5: (No Response)

**********

PLOS authors have the option to publish the peer review history of their article (what does this mean?). If published, this will include your full peer review and any attached files.

If you choose “no”, your identity will remain anonymous but your review may still be made public.

**Do you want your identity to be public for this peer review?**  For information about this choice, including consent withdrawal, please see our Privacy Policy.

Reviewer #1: No

Reviewer #4: **Yes:**  Zhe Wang

Reviewer #5: **Yes:**  James M. Fleckenstein

**Figure resubmission:**

**Reproducibility:** -->To enhance the reproducibility of your results, we recommend that authors of applicable studies deposit laboratory protocols in protocols.io, where a protocol can be assigned its own identifier (DOI) such that it can be cited independently in the future. Additionally, PLOS ONE offers an option to publish peer-reviewed clinical study protocols. Read more information on sharing protocols at https://plos.org/protocols?utm_medium=editorial-email&utm_source=authorletters&utm_campaign=protocols-->?>

# Author comment: Author response to Decision Letter 2

**DOI:** 10.1371/journal.pntd.0014668.r004

21 May 2026

Attachment Submitted filename: ShigellaSerotypingComparison_ResponseReviewers2_may2026.docx

# Peer review: Decision Letter 2

**DOI:** 10.1371/journal.pntd.0014668.r005

**Authors:** Stuart D. Blacksell

12 Aug 2026

Dear Dr Kosek,

We are pleased to inform you that your manuscript 'Robust Performance of Culture, Real-Time PCR, and Genomic Approaches for Shigella Serotyping in a Pediatric Surveillance Cohort' has been provisionally accepted for publication in PLOS Neglected Tropical Diseases.

Before your manuscript can be formally accepted you will need to complete some formatting changes, which you will receive in a follow up email. A member of our team will be in touch with a set of requests.

Please note that your manuscript will not be scheduled for publication until you have made the required changes, so a swift response is appreciated.

IMPORTANT: The editorial review process is now complete. PLOS will only permit corrections to spelling, formatting or significant scientific errors from this point onwards. Requests for major changes, or any which affect the scientific understanding of your work, will cause delays to the publication date of your manuscript.

Should you, your institution's press office or the journal office choose to press release your paper, you will automatically be opted out of early publication. We ask that you notify us now if you or your institution is planning to press release the article. All press must be co-ordinated with PLOS.

Thank you again for supporting Open Access publishing; we are looking forward to publishing your work in PLOS Neglected Tropical Diseases.

Best regards,

Stuart D. Blacksell

Section Editor

PLOS Neglected Tropical Diseases

Shaden Kamhawi

co-Editor-in-Chief

PLOS Neglected Tropical Diseases

orcid.org/0000-0003-4304-636XX

Paul Brindley

co-Editor-in-Chief

PLOS Neglected Tropical Diseases

orcid.org/0000-0003-1765-0002

***********************************************************

Reviewer's Responses to Questions

**Key Review Criteria Required for Acceptance?**

As you describe the new analyses required for acceptance, please consider the following:

**Methods**

-Are the objectives of the study clearly articulated with a clear testable hypothesis stated?

-Is the study design appropriate to address the stated objectives?

-Is the population clearly described and appropriate for the hypothesis being tested?

-Is the sample size sufficient to ensure adequate power to address the hypothesis being tested?

-Were correct statistical analysis used to support conclusions?

-Are there concerns about ethical or regulatory requirements being met?

Reviewer #6: (No Response)

Reviewer #7: (No Response)

**********

**Results**

-Does the analysis presented match the analysis plan?

-Are the results clearly and completely presented?

-Are the figures (Tables, Images) of sufficient quality for clarity?

Reviewer #6: (No Response)

Reviewer #7: (No Response)

**********

**Conclusions**

-Are the conclusions supported by the data presented?

-Are the limitations of analysis clearly described?

-Do the authors discuss how these data can be helpful to advance our understanding of the topic under study?

-Is public health relevance addressed?

Reviewer #6: (No Response)

Reviewer #7: (No Response)

**********

**Editorial and Data Presentation Modifications?**

Use this section for editorial suggestions as well as relatively minor modifications of existing data that would enhance clarity. If the only modifications needed are minor and/or editorial, you may wish to recommend “Minor Revision” or “Accept”.

Reviewer #6: (No Response)

Reviewer #7: (No Response)

**********

**Summary and General Comments**

Use this section to provide overall comments, discuss strengths/weaknesses of the study, novelty, significance, general execution and scholarship. You may also include additional comments for the author, including concerns about dual publication, research ethics, or publication ethics. If requesting major revision, please articulate the new experiments that are needed.

Reviewer #6: (No Response)

Reviewer #7: This manuscript presents a well-designed comparison of culture-based agglutination, real-time PCR, and whole-genome sequencing for Shigella serotyping in a pediatric cohort. The study addresses an important topic, particularly in the context of vaccine development and surveillance. The inclusion of genomic analysis to explain discrepancies between serotyping methods is a major strength and provides valuable biological insight. Overall, the manuscript is well written, scientifically sound, and suitable for publication after minor revision.

1. The authors use manual whole-genome sequence analysis as the reference standard for resolving discrepant results. A brief explanation of why this approach was considered the definitive method would strengthen the manuscript.

2. While overall accuracy is clearly presented, a brief discussion of the sensitivity and specificity results (currently provided in the Supplementary Material) would provide a more balanced assessment of the performance of each method.

3. The study is based on isolates from a single geographic region. Expanding the discussion on how the findings may translate to settings with different circulating Shigella serotypes would improve the interpretation of the results.

**********

PLOS authors have the option to publish the peer review history of their article (what does this mean?). If published, this will include your full peer review and any attached files.

If you choose “no”, your identity will remain anonymous but your review may still be made public.

**Do you want your identity to be public for this peer review?**  For information about this choice, including consent withdrawal, please see our Privacy Policy.

Reviewer #6: **Yes:**  Shumaila Naz

Reviewer #7: **Yes:**  Maqsud Hossain

# Editor report: Acceptance letter

**DOI:** 10.1371/journal.pntd.0014668.r006

**Authors:** Stuart D. Blacksell

Dear Dr Kosek,

We are delighted to inform you that your manuscript, "Robust Performance of Culture, Real-Time PCR, and Genomic Approaches for Shigella Serotyping in a Pediatric Surveillance Cohort," has been formally accepted for publication in PLOS Neglected Tropical Diseases.

We have now passed your article onto the PLOS Production Department who will complete the rest of the publication process. All authors will receive a confirmation email upon publication.

The corresponding author will soon be receiving a typeset proof for review, to ensure errors have not been introduced during production. Please review the PDF proof of your manuscript carefully, as this is the last chance to correct any scientific or type-setting errors. Please note that major changes, or those which affect the scientific understanding of the work, will likely cause delays to the publication date of your manuscript. Note: Proofs for Front Matter articles (Editorial, Viewpoint, Symposium, Review, etc...) are generated on a different schedule and may not be made available as quickly.

Soon after your final files are uploaded, the early version of your manuscript will be published online unless you opted out of this process. The date of the early version will be your article's publication date. The final article will be published to the same URL, and all versions of the paper will be accessible to readers.

For Research Articles, you will receive an invoice from PLOS for your publication fee after your manuscript has reached the completed accept phase. If you receive an email requesting payment before acceptance or for any other service, this may be a phishing scheme. Learn how to identify phishing emails and protect your accounts at https://explore.plos.org/phishing.

Thank you again for supporting open-access publishing; we are looking forward to publishing your work in PLOS Neglected Tropical Diseases.

Best regards,

Shaden Kamhawi

co-Editor-in-Chief

PLOS Neglected Tropical Diseases

Paul Brindley

co-Editor-in-Chief

PLOS Neglected Tropical Diseases
